# Parallel Processing of Olfactory and Mechanosensory Information in the Honey Bee Antennal Lobe

**DOI:** 10.3389/fphys.2021.790453

**Published:** 2021-12-07

**Authors:** Ettore Tiraboschi, Luana Leonardelli, Gianluca Segata, Albrecht Haase

**Affiliations:** ^1^Department of Physics, University of Trento, Trento, Italy; ^2^Center for Mind/Brain Sciences (CIMeC), University of Trento, Rovereto, Italy; ^3^Department of Electrical, Electronic, and Information Engineering, University of Bologna, Bologna, Italy

**Keywords:** mechanosensing, honey bee, antennal lobe, mechanosensory neurons, calcium imaging, *Apis melliera*

## Abstract

In insects, neuronal responses to clean air have so far been reported only episodically in moths. Here we present results obtained by fast two-photon calcium imaging in the honey bee *Apis mellifera*, indicating a substantial involvement of the antennal lobe, the first olfactory neuropil, in the processing of mechanical stimuli. Clean air pulses generate a complex pattern of glomerular activation that provides a code for stimulus intensity and dynamics with a similar level of stereotypy as observed for the olfactory code. Overlapping the air pulses with odor stimuli reveals a superposition of mechanosensory and odor response codes with high contrast. On the mechanosensitive signal, modulations were observed in the same frequency regime as the oscillatory motion of the antennae, suggesting a possible way to detect odorless airflow directions. The transduction of mechanosensory information via the insect antennae has so far been attributed primarily to Johnston’s organ in the pedicel of the antenna. The possibility that the antennal lobe activation by clean air originates from Johnston’s organ could be ruled out, as the signal is suppressed by covering the surfaces of the otherwise freely moving and bending antennae, which should leave Johnston’s organ unaffected. The tuning curves of individual glomeruli indicate increased sensitivity at low-frequency mechanical oscillations as produced by the abdominal motion in waggle dance communication, suggesting a further potential function of this mechanosensory code. The discovery that the olfactory system can sense both odors and mechanical stimuli has recently been made also in mammals. The results presented here give hope that studies on insects can make a fundamental contribution to the cross-taxa understanding of this dual function, as only a few thousand neurons are involved in their brains, all of which are accessible by *in vivo* optical imaging.

## Introduction

Mechanosensory information underlies a variety of behaviors in insects, including negative geotaxis, flight navigation, and social interaction. Among the various mechanosensory receptors all over the insect’s body (Schwartzkopff, 1974), the antennae have been found to play a crucial role. On the antennae, mechanical stimuli are believed to be perceived primarily by mechanosensory neurons in Johnston’s organ (JO), sensitive to movements of the antennal flagellum. These Johnston’s organ neurons (JONs) project into the dorsal lobe (Ai et al., 2009) [in flies also called antennal mechanosensory and motor center (AMMC)]. Their response patterns were shown to provide information on gravity (Kamikouchi et al., 2009), the wind direction (Yorozu et al., 2009; Suver et al., 2019), and for airborne social communication (Clemens et al., 2015; Zhou et al., 2015).

However, in rodents, the involvement of the olfactory system in mechanosensation has recently been discovered. Patch-clamp recording from the olfactory epithelium of mice showed responses of olfactory sensory neurons to mechanical stimuli (Grosmaitre et al., 2007), which was confirmed by calcium imaging of the olfactory bulb (OB) in rats (Carey et al., 2011). A functional role of mechanosensitivity was suggested by experiments showing a reduced odor identification performance by mice in the absence of mechanosensitivity (Iwata et al., 2017). The origin of the signal has been shown to be the same G protein-coupled receptors that detect the odor signal (Connelly et al., 2015).

An identical mechanism in insects is not self-evident, as their olfactory receptors do not belong to the same class, their proteins are inversely oriented within the plasma membrane of the olfactory receptor neurons (ORNs) (Sato et al., 2008). The receptors are housed in sensilla along the antennal flagellum. There are hair-like (sensillum trichodeum type A and B, sensillum basiconicum tick and tapered) and plate-like olfactory sensilla types (sensillum placodeum) (Haase et al., 2011). From the flagellum, ORNs project to the first olfactory neuropil, the antennal lobe (AL), the equivalent to the vertebrate OB.

In the honey bee *Apis mellifera* the antennal lobe consists of 160 network nodes called glomeruli, each invaded by the dendrites of a single class of ORNs only. Glomeruli are interlinked by local neurons and their stereotyped activation patterns encode odor identity and concentration (Galizia et al., 1999b). The glomerular output neurons, called projection neurons (PN) and corresponding to the mitral and tufted cells in vertebrates, send these signals to higher-order brain centers like the mushroom bodies (MBs) and the lateral horns (LHs) (Paoli and Galizia, 2021).

An influence of the airflow on the antennal lobe activity is well-known also in insects, however, only as a carrier of odors, *e.g.*, in studies showing that it enables them to follow odor plumes during navigation (Vickers, 2000) so that the underlying mechanism has been assumed to be a modulation of odor concentration rather than mechanosensitivity.

Antennal lobe responses to clean air have so far been reported only episodically in moths (Kanzaki and Shibuya, 1986; Kanzaki et al., 1989; Park and Cork, 1999; Galizia et al., 2000; Han et al., 2005) until very recently Tuckman et al. (2021a,b) proposed a broader involvement of the olfactory system in mechanosensation based on electrophysiological recordings from single neurons in the moth AL.

To clarify the extent to which the antennal lobe is involved in mechanosensitivity, we systematically investigated glomerular responses during exposure to different airflows with and without additional odor stimuli using two-photon calcium imaging.

## Materials and Methods

### Specimen Preparation for *in vivo* Calcium Imaging

Honey bees were prepared following a well-established protocol (Paoli and Haase, 2018). The bees were exposed to CO_2_ for 30 s. The immobilized bees were then fixed onto a custom-made imaging stage, using soft dental wax (Deiberit 502, Siladent). A small rectangular window was cut into the cuticula above the AL. The glands and trachea covering the AL were moved aside and fura2-dextran, a calcium-sensitive fluorescent dye (Thermo-Fisher Scientific) dissolved in distilled water was injected into the antenno-cerebralis tracts, postero-lateral to the α-lobe using a pulled glass capillary. After the injection, the cuticula was fixed in its original position using n-eicosane (Sigma Aldrich). The bees were stored in a dark, cool, and humid place for 15–20 h to let the dye diffuse into the AL.

Just before the imaging session, antennae were blocked with a drop of n-eicosane on the pedicel leaving the flagellum free to move. The cuticular window, the trachea, and the glands were removed from the antennal lobe region. A silicone adhesive (Kwik-Sil, WPI) was used to cover the brain and a rectangular plastic foil was attached frontal to the window to separate the antennae from the immersion water for the objective lens.

### Two-Photon Microscope

The two-photon microscope (Ultima IV, Bruker) was illuminated by a Ti:Sa laser (Mai Tai Deep See HP, Spectra-Physics). The laser was tuned to 780 nm for fura-2 excitation. All images were acquired with a water immersion objective (10×, NA 0.3, Olympus). Fluorescence was collected in epi-configuration, selected by a dichroic mirror, filtered with a band-pass filter centered at 525 nm and with a 70 nm bandwidth (Chroma Technology), and detected by a photomultiplier tube (Hamamatsu Photonics). The laser power was limited to about 10 mW to reduce photodamage on the specimen, maintaining a good signal-to-noise ratio (SNR) (Paoli et al., 2017a).

### Mechanosensory and Odor Stimulation

The stimulus generator for controlled air and odor pulse delivery was custom built (Supplementary Figure 1). Pure air from a pressure-controlled source passed a charcoal filter and was then humidified by a water flask. The airflow is switched with two solenoid valves in a serial configuration. The first valve opens and closes the airstream. When closed, the airstream is diverted into an exhaust channel to prevent pressure from building up in the system, which creates a rectangular stimulus profile without an initial spike after opening the valve. The second valve determines the flow rate by switching between a large or narrow duct such that the airstream speed can be varied between 1.8 m/s (HF) and 0.25 m/s (LF) and is measured at the position of the antennae with a thermo-anemometer (testo 405i, Testo). A mechanical airflow meter (ANALYT-MTC) is used to adjust the general airflow. Upstream there is a 3-way valve (LHDA0531115, The Lee Company) adding either the oil-immersed odor or pure air to the carrier stream, such that the overall airflow remains constant during the entire stimulation protocol. The airstream is aimed at the bee’s head via a steel tube of 15 cm length and 10 mm cross-section, centered in front of the steel tube is a vertical winglet (10 × 10 mm, L × H) (Supplementary Figure 1). To generate a waggle stimulus, the winglet vibrates laterally, driven by a DC motor to produce a waggle stimulus. The frequency of oscillation is controlled through a PWM pin on the Arduino board. The winglet is coated with aluminum foil and grounded to earth to prevent electrostatic charges in the airstream. The distance between the winglet tip and the head of the bee is about 15 mm. The solenoid valves and the DC motor are controlled with an Arduino Uno board (Arduino) through custom software. Sound stimuli were generated using the Arduino Uno board, an audio amplifier board module (HiLetgo TDA2822M), and a speaker of 28 mm diameter, 8 Ω, and 2 W placed 15 mm frontally to the bee. Stimulation protocols were generated through MATLAB (R2019b, MathWorks) scripts and delivered to the Arduino board through a PCIe-6321 multifunction board (National Instruments). A recording session started with 10 s of background signal acquisition followed by alternating different types of stimuli in a pseudorandom order up to 15 trials per stimulus. The duration of the main mechanical stimulus was 6 s, during which an airstream of different intensities, LF (0.25 m/s), HF (1.8 m/s), and no-air (0 m/s), was delivered. In the middle of this time window, a secondary stimulus of 3 s could be added (waggling Wag or odor 3Hex). The main stimulus period is followed by an interstimulus interval of 6 s. For the odor stimulus, 3-hexanol (W335118, Sigma-Aldrich) was used, diluted 1:25 in mineral oil. Only the head of the bee is exposed to air/odor stimuli as the body is enclosed in the mounting stage to minimize mechanosensory stimulation of the insect body.

### Image Acquisition

The image acquisition was synchronized to the stimulus protocol at a frame rate of 10.083 fps. The image of 128 × 128 pixels with a digital zoom factor of 3.8 covers a field of view of 280 μm. The fluorescence intensity was recorded with a depth of 13 bits. In addition to the functional images, a *z*-stack of the antennal lobe was acquired at a spatial resolution of 512 × 512 pixels and a layer interval of 2 μm to perform the morphological identification of glomeruli.

### Image Analysis

A total of 7 bees were recorded and analyzed. Data post-processing and analysis were performed employing custom scripts in MATLAB. In each bee, the recorded glomeruli were identified using the AL atlas (Galizia et al., 1999a) and associated with regions of interest (ROI) over which the fluorescence signals were spatially averaged. From these raw data the relative change of fluorescence during the stimulus expressed in%: Δ*F*/*F* = −[*F*(t) − *F*_*b*_]/*F*_*b*_ × 100, where *F*_*b*_ is the average fluorescence signal in the 3 s pre-stimulus period. This is a measure for the neuronal firing rate (Moreaux and Laurent, 2007) in each glomerulus. Finally, for each stimulus, Δ*F*/*F* was averaged over the 15 trials to obtain the mean response for each glomerulus to a stimulus.

To identify glomeruli with the highest variance during the stimuli, a PCA was performed on the pixels as variables with frames as observations (Supplementary Figure 2).

### Statistical Analysis of the Stereotypy

The dependencies of the glomerular responses were tested in a repeated-measures ANOVA on each of the 17 glomeruli to which at least two bees contributed (Supplementary Figure 3). Bees entered as subjects and stimulus type and trials as within-subject factors.

**FIGURE 1:**
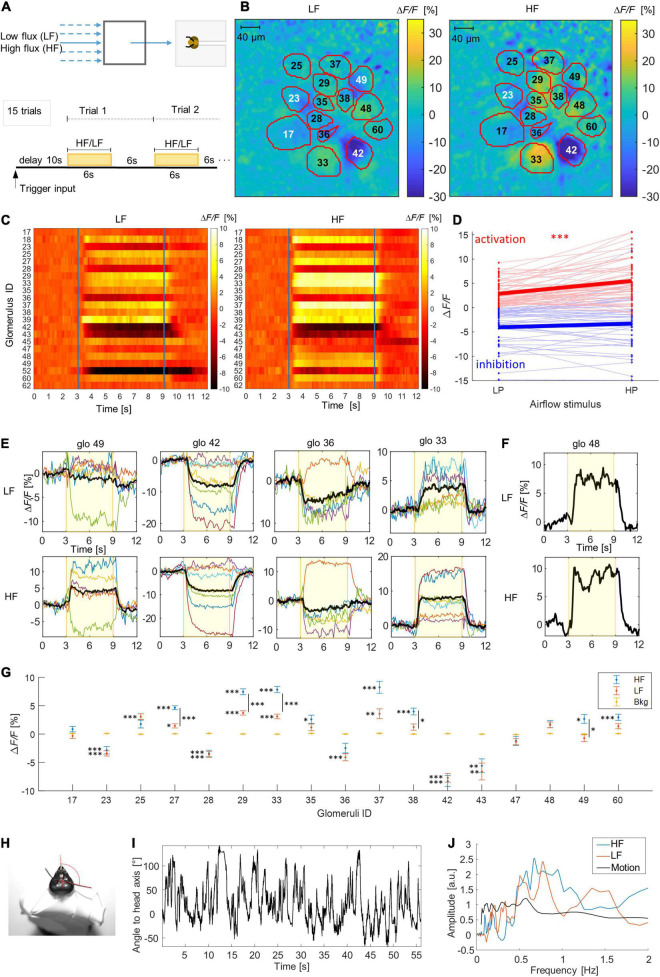
Response patterns to airflow stimuli. **(A)** Setup scheme and stimulus protocol. Stimuli start after 10 s of background acquisition, lasting 6 s (yellow area), inter-trial distance 6 s. **(B)** Example for the relative fluorescence change (Δ*F*/*F* [%]) in the imaging plane across the AL. Outlines and labels show the identified glomeruli. **(C)** Heatmaps show subject-averaged (*N* = 7) responses of all imaged glomerulus to low flux (LF) and high flux (HF) delivered after 3 s. **(D)** Change of the glomerular activation between LF and HF, activated glomeruli (red dots) increase responses significantly [paired *t*-test: *t*(56) = –5.51, *p* = 10^– 7^], inhibited glomeruli (blue dots) don’t [*t*(49) = –1.65, *p* = 0.11]. **(E)** Temporal response curves of four selected glomeruli to LF and HF airflow. Colored curves show single subject responses, averaged over 15 trials. The black curve is the subject-averaged response. The yellow background marks the stimulus interval. **(F)** Example of glomeruli showing an oscillatory modulation of the activity signal. **(G)** Glomerular response amplitudes during the central 3 s of stimuli with different airflow intensities. Shown are mean values and standard errors across all trials of all bees (blue: high flux, red: low flux, yellow: background signal). Significant differences with respect to the background are marked with ^∗^*p* < 0.05, ^∗∗^*p* < 0.01, ^∗∗∗^*p* < 0.001. Significant differences between different flux velocity responses are marked with an additional vertical bar. Full statistical results are shown in Supplementary Table 2. **(H)** Bee mounted with the head and the antennae free to move for high-speed antenna motion imaging, current angle of the right flagellum is marked in red. **(I)** Example for an antenna tracking curve during 1 min of recording. **(J)** Averaged spectra of the oscillatory activity in panel **(F)** (red LF, blue HF) and spectrum of the antenna motion in panel **(I)**.

**FIGURE 2:**
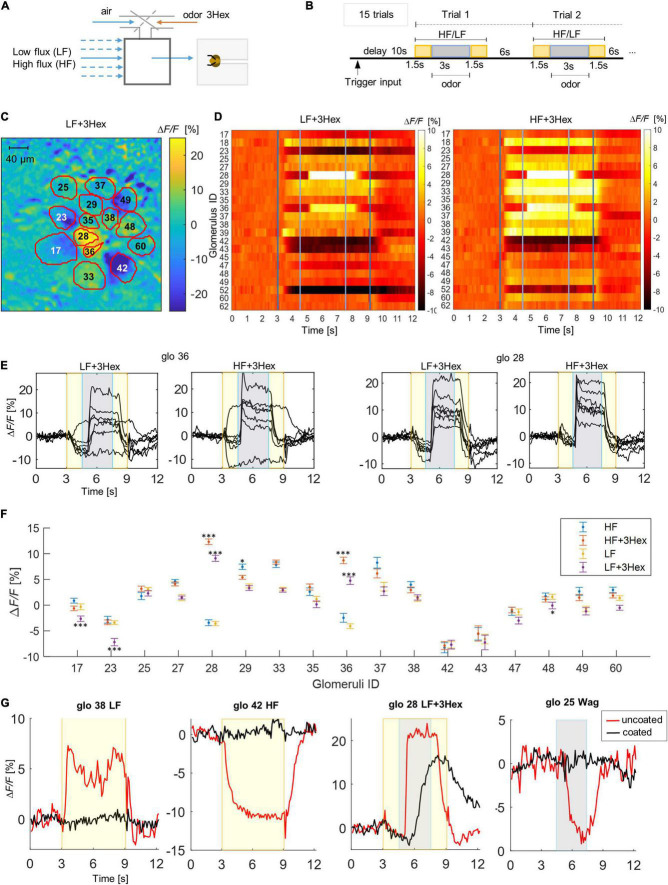
Response patterns to mechanical and odor stimuli. **(A)** Scheme of the setup where either clean air or 3-hexanol (3Hex) is injected into the carrier flux. **(B)** Scheme of the stimulation protocol: To the airflow stimulus starting after 10 s (yellow area), the 3-Hex odor stimulus is added after another 1.5 s lasting 3 s (gray area), interstimulus interval 6 s. **(C)** Relative fluorescence change in the imaging plane during the low air flux plus odor stimulus (LF + 3Hex), outlines and labels show the identified glomeruli. **(D)** Heatmaps show subject-averaged responses of all imaged glomerulus to LF and HF delivered after 3 s and air plus odor after 4.5 s. **(E)** Temporal response curves of the two glomeruli (28,36) that showed responses to the odor stimulus. Shown are responses of the seven different bees averaged across the 15 trials. Yellow areas mark the air only periods, gray boxes the air plus odor periods. **(F)** Mean values and Standard errors across bees of the glomerular response amplitudes during the central 3 s of a stimulus with and without odor (blue: high flux only, red: high flux + 3-Hexanol, yellow: low flux only, violent: low flux + 3-Hexanol). Significant differences between air only and air + odor stimuli are marked with ^∗^*p* < 0.05, ^∗∗∗^*p* < 0.001. Full statistical results are shown in Supplementary Table 3. **(G)** Temporal response curves of selected glomeruli to weak flow (LF), HF, weak flow + odor (LF + 3Hex), and waggling (Wag) for bees with antennae coated with fluid silicon (black) and uncoated antennae (red).

**FIGURE 3:**
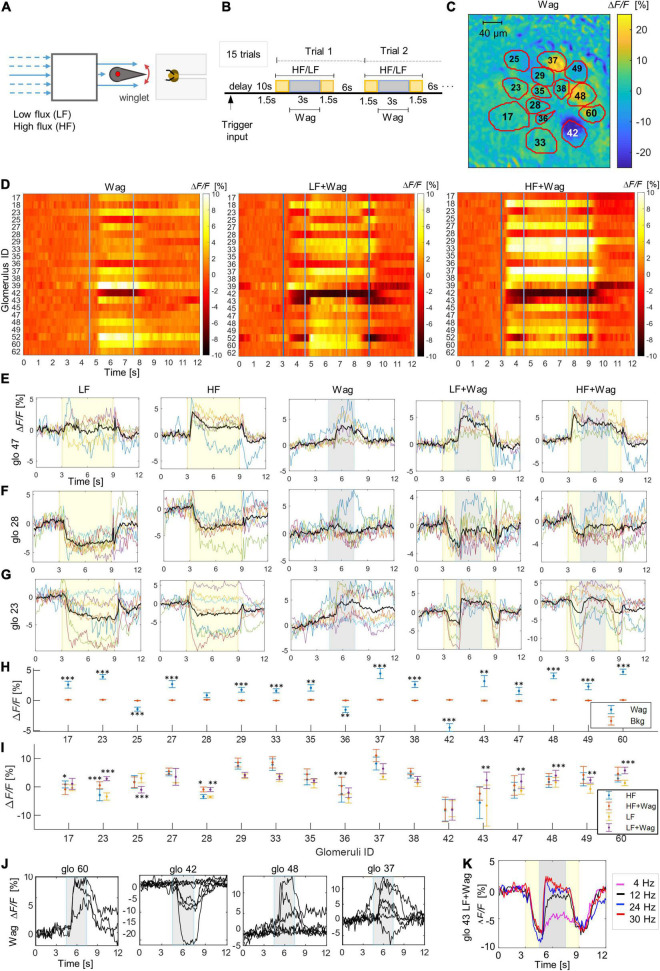
Response patterns to waggle motion. **(A)** Stimulus generator scheme for laminar airflow and waggle-dance-like stimuli via an oscillating winglet. **(B)** Stimulation protocol with laminar flow stimuli starting after 10 s of background acquisition, lasting 6 s (yellow area) and a waggle motion added to it after 1.5 s lasting 3 s (gray areas), inter-stimulus interval 6 s. **(C)** Relative fluorescence change in the imaging plane during stimulus only by the waggle motion (Wag) without additional airflow, outlines and labels show the identified glomeruli. **(D)** Heatmaps show the subject-averaged glomerular responses to the waggle only stimulus (Wag) and combined stimuli where waggling is added after 4.5 s to the low flux (LF + Wag) or the high flux (HF + Wag). **(E)** Temporal response curves to single and combined stimuli of glomerulus 47 which is sensitive already to waggling only. **(F)** Temporal response curves to single and combined stimuli of glomerulus 28 which is not sensitive to waggling only, where waggling stronger modulates the LF stimulus. **(G)** Temporal response curves to single and combined stimuli of glomerulus 23 sensitive to waggling and where waggling stronger modulates the HF stimulus. **(H)** Mean values and standard errors across trials and bees of the glomerular responses during the central 3 s of a waggling only stimulus with respect to the background signal. Significant differences between waggling and the background signal are marked with ^∗^*p* < 0.05, ^∗∗^*p* < 0.01, ^∗∗∗^*p* < 0.001. Full statistical results are shown in Supplementary Table 4. **(I)** Mean values and standard errors across trials and bees of the glomerular responses during the central 3 s of the two airflow strength signals with and without additional waggling (blue: high flux only, red: high flux and waggling, yellow: low flux only, violent: low flux and waggling). Significant differences between air only and air + waggling are marked with *p < 0.05, **p < 0.01, ***p < 0.001. Full statistical results are shown in Supplementary Table 5. **(J)** Temporal response curves of 4 selected glomeruli to waggle motion only. **(K)** Response of a selected glomerulus to a low flux stimulus with superimposed waggle motion at different frequencies.

A series of multiple comparisons was used to test the stereotypy of the glomerular responses across bees for the different stimulus types and a prestimulus background signal. The bee-and trial-averaged responses during the central 3 s of the experiment (Figures 1B, 2A, 3B) were confronted between the stimulus types of interest within each glomerulus by paired *t*-tests. A Bonferroni-Holm correction protected the results from type I errors.

Furthermore, a principal component analysis was performed on the full dataset using as features the averaged glomerular response in the first 1.5 s of each stimulus for the LF *vs.* HF *vs.* no-air comparison whereas for the LF *vs.* LF-3Hex the average over the last 2 s of the odor stimulus was used. The glomerular responses for no-air were computed averaging over 1.5 s before the stimulus. Every single recording corresponds to an observation. Statistical differences in the distribution of each group were evaluated using the statistical energy test (Aslan and Zech, 2005). The multiple comparisons were again corrected via the Bonferroni-Holm method.

### Antenna Tracking

The antenna motion was recorded with a JVC GC-PX100BE Camcorder. A frame rate of 200 Hz turned out to be the best compromise between temporal and spatial resolution (640 × 360 pixels). Recordings were analyzed via custom python scripts. Images were background subtracted and binarized, and the antennae were identified via connected component analysis. Antenna images were then skeletonized, and the flagellum axis was obtained via the Hough transform. Its angle was measured against the head axis, which was obtained by polygonal fitting the head contour.

## Results

### Clean Air Stimuli

The clean air stimuli (Supplementary Figure 1) were generated at two specific airflow velocities, one that a bee would typically experience during flight (high flux, HF = 1.8 m/s) and one that wing beating would produce during the waggle dance (low flux, LF = 0.25 m/s) (Michelsen, 2003).

Exposed to repeated airflow stimuli (Figures 2A,B), clear and consistent responses were observed in most of the imaged glomeruli (Figures 2C,D and Supplementary Video 1). A repeated-measures ANOVA showed a significant dependence on the stimulus type for 11 of the 17 analyzed glomeruli, but no significant dependence on the trails in all but one glomeruli, reflecting the high reproducibility of the results within bees (Supplementary Table 1).

But beyond previously reported activation (Tuckman et al., 2021a), also strong inhibition was found in several glomeruli (Figures 2C,E). This suggests that the mechanosensitivity of receptor neurons is non-uniform and that probably the same inhibitory local neurons involved in odor coding generate these combinatorial patterns encoding airflow stimuli.

To test the code’s stereotypy, the individual glomeruli were identified via the AL atlas (Galizia et al., 1999a) and the experiment was repeated in 7 subjects. Results show that the preservation of the response patterns across individuals is as high as for the odor code (Galizia et al., 1999b). Responses in exemplary glomeruli (Figure 2E) shows only rare deviations in single bees. A statistical analysis demonstrates that bee- and trial-averaged responses are significantly different from the background signal in all but 3 of the tested glomeruli for at least one of the airflow velocities (LF/HF), consistently for both inhibited and excitatory responses. In 5 glomeruli also the difference between LF and HF is significant (Figure 2G and Supplementary Table 2). The number of bees that contribute to each of that mean glomerular responses together with the individual amplitudes can be found in Supplementary Figure 3. A PCA analysis of the multidimensional coding space confirms this clear deviation from the background and a clustering of both LF and HF responses from different bees along PC2 (Supplementary Figure 4A). Both effects are significant under a statistical energy test.

Confronting single glomerular responses to both fluxes shows that on average the glomerular activation amplitude is proportional to the airflow intensity in case of excitatory responses, glomerular inhibition remained constant (Figure 2D). The glomerular response to the airflow rarely attenuates during the 6 s of exposure (Figure 2E), in contrast to odor responses which often decrease over time.

A particular case of signal modulation is shown in Figure 2F, where the glomerular response shows an oscillatory modulation, which is consistently reproduced during the 15 trials. Comparing this modulation with the angular motion of the flagellum, obtained by high-speed video tracking (Figures 2H,I), shows that both signals are in the same frequency range with major components between 0.5 and 1 Hz (Figure 2J).

### Air and Odor Stimuli

Next, responses elicited by a superposition of mechanical and odor stimuli were tested by injecting 3-Hexanol (3Hex) into the air stream (Figures 1A,B), an odor that is known to excite glomeruli 28 and 36 (Paoli et al., 2018). In this experiment, the odor stimulus lasted 3 s and was added after 1.5 s to the airflow stimulus, without changing the overall flux (Figures 1C,D). Both glomeruli, which are initially inhibited by the airflow, show a reversal of this inhibition into a strong activation (Figures 1C–E and Supplementary Video 2). A multiple comparison analysis between both stimuli in all glomeruli, confirmed the highly significant changes in the two glomeruli known to be addressed by 3-Hexanol. Additional significant inhibitive changes were observed in glomeruli 17 and 23 (Figure 1F and Supplementary Table 3).

### Coated Antennae

To verify the origin of both signals, the flagella of three of the imaged bees were coated with a thin layer of silicone, leaving them free to move. The observed mechanosensory response was now highly attenuated, whereas the odor response was as strong as before although with slower response dynamics (Figure 1G).

### Waggle Motion Stimuli

Next, a potential role of the AL mechanosensation in the waggle dance communication was tested, where dancer bees communicate angle and distance of a food source by wing beating and abdominal oscillations to dance followers (Ai et al., 2009). Michelsen (2003) reported that the airflow elicited by the wing beating had velocities from 0.15 to 0.3 m/s and was modulated by abdominal oscillations at a frequency of 24–25 Hz. A waggle-dance-like stimulus (Wag) was produced by oscillating a winglet at 24 Hz in a laminar airflow of 0.25 m/s (Figures 3A,B). Already the oscillating winglet by itself (without additional airflow) elicited a stereotypical response, that was statistically significant in all but one glomeruli (Figure 3H and Supplementary Table 4), either activating or inhibiting them (Figures 3C,D and Supplementary Video 3). The airflow generated by the winglet is very weak (average speed 0–0.03 m/s), however, being very turbulent, it may generate strong local gradients leading to a pulsed-like stimulation of vibrational movements of the hair-like sensilla. Embedding the waggle stimulus in a laminar airflow, reproduces precisely the airflow felt by a bee that is following the dancer. The results clearly show that this modulation of the laminar airflow is effectively detected by the glomeruli (Figures 3C,D and Supplementary Video 3). The waggle stimulus is more effective in a slow airflow and shows different characteristics in the glomerular responses. Most glomeruli were found to be sensitive already to the waggle stimulus without additional airstream (Figures 3E,G,J), others were sensitive only to a combined waggling/airflow stimulus (Figure 3F). Some were tuned to detect waggling in a weak flow but not in the strong flow (Figure 3E) and others again were modulated in a weak and strong flow (Figures 3F,G). A statistical analysis showed significant changes due to waggling within a low flux airstream in 8 glomeruli. Within a high flux airstream, waggling induced significant changes in 4 glomeruli (Figure 3I and Supplementary Table 5). This rich repertoire of responses suggests a high dynamic range of the mechanoreception mechanism which would allow for coding of complex temporal patterns (Supplementary Figures 5, 6 show the complete spatio-temporal response pattern to all stimuli in a representative bee).

The dependence of the glomerular activity on the modulation frequency of the airflow was tested by a variation of the winglet oscillation. Experiments were repeated with a weak airflow and oscillation frequencies of 4, 12, 24, and 30 Hz. The results show that the modulatory effect saturates at 24 Hz with no further increase at 30 Hz, while at lower frequencies the effect decreases proportionally (Figure 3K and Supplementary Video 4).

Since a bee during waggle dance also produces oscillations at higher frequencies around 200–400 Hz via wing beating, the sensitivity of the AL also to these signals was tested by exposing the bees to comparable stimuli produced by a loudspeaker ramping frequency from 40 up to 6,000 Hz. No glomerular response to any of these stimuli was observed.

## Discussion

Until now, the honey bee antennal lobe was thought to be activated only by olfactory stimuli. The influence of airflow on the responses was exclusively attributed to a modulation of the odor concentration. The data presented here show that airflow stimulates the antennal lobe even in the absence of odorants. The extent of the responses, involving almost all glomeruli, is a fundamental difference from the sparse odor response patterns. This confirms the corresponding conclusions of Tuckman et al. (2021a) based on electrophysiological recordings in moths. This wide spectrum of responses together with the large response amplitudes excludes the possibility that they originate from residual odorous substances in the purified air pulses. Furthermore, the fact that the simple winglet oscillation generates a broad response in most glomeruli excludes that odor contaminants produce the responses, as no air stream is added in this case.

The complexity of the response patterns ranging from high excitation to complete inhibition of projection neuron activity suggests that this is not a simple background effect without functional relevance. The highly dynamic nature of the signal and, above all, the stereotypy of the combinatorial code makes it likely that the encoded information is used, *e.g.*, to detect wind speed and direction during flight. One of the two airflow velocities (HF = 1.8 m/s) was chosen to resemble typical flight speeds, so the high sensitivity to this stimulus underlines the suitability for this purpose. The tonic nature of the mechanosensitive responses suggests that glomeruli may record wind speed persistently during flight. Regarding the tonic character of the mechanosensitive responses, our results deviate from those of Tuckman et al. (2021a) who reported a generally fast decaying response to air stimuli, but we doubt that their generalization is permissible given that the experimental data are based on air stimuli that lasted only 200 ms.

The oscillatory modulations on the mechanosensory responses were found to be in the same frequency window as the azimuthal motion of the antennae that was measured by a high-speed camera. From a physical point of view, the reorientation of the flagella would change the direction under which the airflow hits the hair-like sensilla and would strongly modulate their degree of motion. It is the projection angle between wind direction and sensillum axis, that quantifies the strength of the force acting on it, reaching from zero when sensilla that are aligned in parallel and downwind to a maximum for orthogonal orientation. This seems to produce a direction-dependent signal modulation. Bees might use this direction sensitivity not only to detect odor gradients, as cockroaches do via position-dependent activation along the antennae (Nishino et al., 2018), but also to sample the wind direction during flight.

The simultaneous presentation of an odor stimulus with the air pulse showed that the chemical signal was perceived without a loss of contrast nor of dynamic range compared to experiments where instead of an air pulse a continuous carrier stream is used (Paoli et al., 2018). On the contrary, the observed inhibitive changes due to 3-Hexanol in two glomeruli were not evident in previous experiments and might indicate a certain increase of sensitivity of the system by an underlying tonic signal. But this effect disappeared at higher fluxes, where the excitatory mechanical response seems to dominate. At realistic airflow velocities, chemosensation was found to be dominant, which can be expected since airflow is a rather continuous stimulus during straight flight, whereas olfactory stimuli are sparse, highly variant, and of great relevance and therefore require precedence in perception. The fact that responses to odor stimuli involve only a much smaller proportion of the glomeruli (Galizia et al., 1999b) and the usually transient dynamics of PN responses to olfactory stimuli (Mazor and Laurent, 2005) might form the basis for selective decoding of both types of signals in higher-order brain centers.

The experiments with bees whose antenna surfaces were coated with silicon, without restricting the flagella movement, showed that odor responses were temporally delayed and mechanosensory responses strongly suppressed. This is a further argument against the possibility that the responses to the air pulses originate from odor impurities, which in that case should be equally delayed but not suppressed. It also suggests that the sources for the mechanosensory signal are likely located in the olfactory sensilla hairs on the antenna surface and are activated by changes in sensilla position, motion, or shape. The resulting mechanical forces may cause conformational changes directly in the olfactory receptors and/or could modulate an electrical coupling between different olfactory neurons within one sensillum (Vermeulen and Rospars, 2004) by changing their distance. The latter would rather explain a modulation of the olfactory signal. Sensilla of varying lengths and sections would react differently under specific airstream intensity, thus broadening a response spectrum, or even tuning to very specific motion parameters like oscillatory frequencies.

This motion was strongly damped by the silicon coating and accordingly, there was no mechanical activation. Odor molecules were nevertheless able to diffuse toward the chemoreceptors although with strongly reduced velocities, which explains the delay in odor response signals. If the origin of the antennal lobe activation were Johnston’s organ neurons, a response to mechanical stimuli should have been observed, as the flagellum was free to move, and the stretch-sensitive JONs were hardly affected by the coating.

Alternative sensory modalities such as hygro- or thermosensation are highly unlikely to be the origin of this signal. Apart from the fact that such sensory modalities have never been reported from the honey bee antennal lobe, in cockroaches and Drosophila, only a few peripheral glomeruli showed hygro- or thermosensitivity (Nishino et al., 2003; Enjin et al., 2016), whereas here the majority of glomeruli responded.

When adding a periodic low-frequency modulation to the airflow the sensitivity to the signal was found to increase in several glomeruli. The frequency dependence of this effect suggests that this might be tuning to frequencies as they occur during the waggle dance communication. The response to oscillations reaches a maximum at 24 Hz, a frequency that was reported to provide the most efficient transfer of information during the waggle dance (Michelsen, 2003). This frequency is perceived by follower bees that are aligned within 30° to the dancer bee’s body axis. If instead the receiver bee is located more laterally to the dancer, the perceived oscillation frequency drops by one half to ca. 12 Hz and the information transfer was found to be less effective (Michelsen, 2003). Also in this study, the response to airflow modulations at 12 Hz was found to be strongly reduced. This enhanced frequency sensitivity suggests another potential purpose of the mechanosensitive response. It allows the detection of the abdominal waggle motion, suggesting a further pathway for information transfer on the complex dancing pattern that encodes the numerical information on the direction and distance of a food source. So far it was shown that Johnston’s organ is most certainly involved in waggle motion detection, but neurons seem to be tuned to 230–265 Hz, the wing beating frequency during waggle dance (Ai et al., 2009; Greggers et al., 2013). It is therefore possible that the antennal lobe provides complementary information focusing on the slower abdominal motion.

In summary, these results provide the first evidence for parallel coding of odor and mechanical stimuli in the honey bee AL. In contrast to previous studies that have suggested Johnston’s organ as the primary organ for antennal mechanoreception, the here provided data suggest that the glomerular response code might contribute considerably to it, especially at lower frequencies corresponding to modulation of wind direction during flight or abdominal motion during waggle dance.

This study suggests that our view on the insect olfactory system, which has expanded considerably over the last decades (Pannunzi and Nowotny, 2019), needs to be revised once again, as it appears to be involved in processing an even wider range of airborne stimuli. This also shows further similarities to the mammalian system, where mechosensitivity has recently been discovered. The broad involvement of sensory neurons overserved here is equivalent to findings in mice, where 70% of the sensory neurons in the septal organ and 50% in the main olfactory epithelium showed mechanosensitivity (Grosmaitre et al., 2007), as well as in rats, where 50% of all glomeruli of the OB showed significant odorant-free responses (Carey et al., 2011). Although the neuronal organization of the peripheral olfactory system in mammals is very similar to those of insects, there are substantial differences in their location. Olfactory receptors are sitting in cilia hairs on the surface of the epithelium of the nasal cavity. They are not in direct contact with air but covered by a mucus layer. However, this mucus is in constant motion driven by ciliary beating but also by the airflow through the nasal cavity. Olfactory cilia, lacking a dynein arm, are not involved in this active beating (Menco, 1984), they are, however, exposed to the forces of the motion of the mucus and thus directly influenced by external airflow changes (Wakazono et al., 2017). This would allow the mechanosensitive responses in mammals and insects to have the same origin.

Also the additive nature of the mechanical and the olfactory code confirms findings in mice. Air pulses enhanced the firing frequency of individual neurons weakly stimulated by odorants (Grosmaitre et al., 2007). This might contribute to the exceptional odor sensitivity of bees (Paoli et al., 2017b) and mice (Dewan et al., 2018), because even if a continuous air stream adds a tonic random background to an odor pulse, the mechanism of stochastic resonances may enhance the information transduction from the odor signal. This has been shown in various sensory modalities, *e.g.*, mechanoreception in crayfish (Douglass et al., 1993) and crickets (Levin and Miller, 1996), in the hippocampal network of mice (Gluckman et al., 1996), and in visual perception in humans (Piana et al., 2000). A first indication for such an increase of sensitivity might be the two inhibited glomerular responses to 3-Hexanol that were not visible in previous experiments (Paoli et al., 2018).

Along the same line, Iwata et al. (2017) found that mechanosensitivity enhances rather than masks odors signals in the mice OB regarding the temporal code, a coding modality also observed in the honey bee antennal lobe (Paoli et al., 2018).

In conclusion, one can safely say that dual coding of odors and mechanical stimuli is another of the many conserved features of the olfactory system between insects and mammals (Ache and Young, 2005). This provides new arguments for the importance of the honey bee as a neuroethological model, as there is a legitimate hope that studies on a network of a few thousand neurons will contribute significantly to the understanding of the role of mechanosensation in the olfactory system as well as the underlying mechanisms.

## Data Availability Statement

The raw data supporting the conclusion of this article will be made available by the authors, without undue reservation.

## Author Contributions

ET designed the study, developed the methodology, acquired and analyzed the data. LL acquired the data and contributed to the data analysis. GS analyzed the antenna motion data. AH contributed to the data analysis and provided the funding and instrumentation. All authors contributed to the preparation of the manuscript.

## Conflict of Interest

The authors declare that the research was conducted in the absence of any commercial or financial relationships that could be construed as a potential conflict of interest.

## Publisher’s Note

All claims expressed in this article are solely those of the authors and do not necessarily represent those of their affiliated organizations, or those of the publisher, the editors and the reviewers. Any product that may be evaluated in this article, or claim that may be made by its manufacturer, is not guaranteed or endorsed by the publisher.

## References

[B1] Ache B. W. Young J. M. (2005). Olfaction: diverse species, conserved principles. *Neuron* 48 417–430. 10.1016/j.neuron.2005.10.022 16269360

[B2] Ai H. Rybak J. Menzel R. Itoh T. (2009). Response characteristics of vibration-sensitive interneurons related to Johnston’s organ in the honeybee, *Apis mellifera*. *J. Comp. Neurol.* 515 145–160. 10.1002/cne.22042 19412925

[B3] Aslan B. Zech G. (2005). Statistical energy as a tool for binning-free, multivariate goodness-of-fit tests, two-sample comparison and unfolding. *Nucl. Instruments Methods Phys. Res. Sect. A Accel. Spectrometers Detect. Assoc. Equip.* 537 626–636. 10.1016/j.nima.2004.08.071

[B4] Carey R. M. Verhagen J. V. Wesson D. W. Pírez N. Wachowiak M. Frank M. E. (2011). Temporal structure of receptor neuron input to the olfactory bulb imaged in behaving rats. *Stimulus* 101 1073–1088. 10.1152/jn.90902.2008 19091924 PMC2657066

[B5] Clemens J. Girardin C. C. Coen P. Guan X. J. Dickson B. J. Murthy M. (2015). Connecting neural codes with behavior in the auditory system of *Drosophila*. *Neuron* 87 1332–1343. 10.1016/j.neuron.2015.08.014 26365767 PMC4629847

[B6] Connelly T. Yu Y. Grosmaitre X. Wang J. Santarelli L. C. Savigner A. (2015). G protein-coupled odorant receptors underlie mechanosensitivity in mammalian olfactory sensory neurons. *Proc. Natl. Acad. Sci. U. S. A.* 112 590–595. 10.1073/pnas.1418515112 25550517 PMC4299258

[B7] Dewan A. Cichy A. Zhang J. Miguel K. Feinstein P. Rinberg D. (2018). Single olfactory receptors set odor detection thresholds. *Nat. Commun.* 9:2887. 10.1038/s41467-018-05129-0 30038239 PMC6056506

[B8] Douglass J. K. Wilkens L. Pantazelou E. Moss F. (1993). Noise enhancement of information transfer in crayfish mechanoreceptors by stochastic resonance. *Nature* 365 337–340. 10.1038/365337a0 8377824

[B9] Enjin A. Zaharieva E. E. Frank D. D. Mansourian S. Suh G. S. B. Gallio M. (2016). Humidity sensing in *Drosophila*. *Curr. Biol.* 26 1352–1358. 10.1016/j.cub.2016.03.049 27161501 PMC5305172

[B10] Galizia C. G. Sachse S. Rappert A. Menzel R. (1999b). The glomerular code for odor representation is species specific in the honeybee *Apis mellifera*. *Nat. Neurosci.* 2 473–478. 10.1038/8144 10321253

[B11] Galizia C. G. McIlwrath S. L. Menzel R. (1999a). A digital 3-dimensional atlas of the honeybee antennal lobe based on optical sections acquired using confocal micoscropy. *Cell Tissue Res.* 295 383–394. 10022959 10.1007/s004410051245

[B12] Galizia C. G. Sachse S. Mustaparta H. (2000). Calcium responses to pheromones and plant odours in the antennal lobe of the male and female moth *Heliothis virescens*. *J. Comp. Physiol. A.* 186 1049–1063. 10.1007/s003590000156 11195281

[B13] Gluckman B. J. Netoff T. I. Neel E. J. Spano W. L. Spano M. L. Schiff S. J. (1996). Stochastic resonance in a neuronal network from mammalian brain. *Phys. Rev. Lett.* 77 4098–4101. 10.1103/PhysRevLett.77.4098 10062387

[B14] Greggers U. Koch G. Schmidt V. Durr A. Floriou-Servou A. Piepenbrock D. (2013). Reception and learning of electric fields in bees. *Proc. R. Soc. B Biol. Sci.* 280:20130528. 10.1098/rspb.2013.0528 23536603 PMC3619523

[B15] Grosmaitre X. Santarelli L. C. Tan J. Luo M. Ma M. (2007). Dual functions of mammalian olfactory sensory neurons as odor detectors and mechanical sensors. *Nat. Neurosci.* 10 348–354. 10.1038/nn1856 17310245 PMC2227320

[B16] Haase A. Rigosi E. Frasnelli E. Trona F. Tessarolo F. Vinegoni C. (2011). A multimodal approach for tracing lateralisation along the olfactory pathway in the honeybee through electrophysiological recordings, morpho-functional imaging, and behavioural studies. *Eur. Biophys. J.* 40 1247–1258. 10.1007/s00249-011-0748-6 21956452 PMC3366498

[B17] Han Q. Hansson B. S. Anton S. (2005). Interactions of mechanical stimuli and sex pheromone information in antennal lobe neurons of a male moth, *Spodoptera littoralis*. *J. Comp. Physiol. A Neuroethol. Sensory, Neural, Behav. Physiol.* 191 521–528. 10.1007/s00359-005-0618-8 15856257

[B18] Iwata R. Kiyonari H. Imai T. (2017). Mechanosensory-Based Phase Coding of Odor Identity in the Olfactory Bulb. *Neuron* 96 1139–1152.e7. 10.1016/j.neuron.2017.11.008 29216451

[B19] Kamikouchi A. Inagaki H. K. Effertz T. Hendrich O. Fiala A. Göpfert M. C. (2009). The neural basis of *Drosophila* gravity-sensing and hearing. *Nature* 458 165–171. 10.1038/nature07810 19279630

[B20] Kanzaki R. Arbas E. A. Strausfeld N. J. Hildebrand J. G. (1989). Physiology and morphology of projection neurons in the antennal lobe of the male mothManduca sexta. *J. Comp. Physiol. A* 165 427–453. 10.1007/BF00611233 2769606

[B21] Kanzaki R. Shibuya T. (1986). Descending protocerebral neurons related to the mating dance of the male silkworm moth. *Brain Res.* 377 378–382. 10.1016/0006-8993(86)90885-1 3730870

[B22] Levin J. E. Miller J. P. (1996). Broadband neural encoding in the cricket cercal sensory system enhanced by stochastic resonance. *Nature* 380 165–168. 10.1038/380165a0 8600392

[B23] Mazor O. Laurent G. (2005). Transient dynamics versus fixed points in odor representations by locust antennal lobe projection neurons. *Neuron* 48 661–673. 10.1016/j.neuron.2005.09.032 16301181

[B24] Menco B. P. (1984). Ciliated and microvillous structures of rat olfactory and nasal respiratory epithelia - A study using ultra-rapid cryo-fixation followed by freeze-substitution or freeze-etching. *Cell Tissue Res.* 235 225–241. 10.1007/BF00217846 6367994

[B25] Michelsen A. (2003). Signals and flexibility in the dance communication of honeybees. *J. Comp. Physiol. A Neuroethol. Sensory, Neural, Behav. Physiol.* 189 165–174. 10.1007/s00359-003-0398-y 12664092

[B26] Moreaux L. Laurent G. (2007). Estimating firing rates from calcium signals in locust projection neurons in vivo. *Front. Neural Circ.* 1:2. 10.3389/neuro.04.002.2007 18946544 PMC2526277

[B27] Nishino H. Iwasaki M. Paoli M. Kamimura I. Yoritsune A. Mizunami M. (2018). Spatial receptive fields for odor localization. *Curr. Biol.* 28 600–608.e3. 10.1016/j.cub.2017.12.055 29429617

[B28] Nishino H. Yamashita S. Yamazaki Y. Nishikawa M. Yokohari F. Mizunami M. (2003). Projection neurons originating from thermo- and hygrosensory glomeruli in the antennal lobe of the cockroach. *J. Comp. Neurol.* 455 40–55. 10.1002/cne.10450 12454995

[B29] Pannunzi M. Nowotny T. (2019). Odor stimuli: not just chemical identity. *Front. Physiol.* 10:1428. 10.3389/fphys.2019.01428 31827441 PMC6890726

[B30] Paoli M. Albi A. Zanon M. Zanini D. Antolini R. Haase A. (2018). Neuronal response latencies encode first odor identity information across subjects. *J. Neurosci.* 38 9240–9251. 10.1523/jneurosci.0453-18.2018 30201774 PMC6705991

[B31] Paoli M. Andrione M. Haase A. (2017a). “Imaging techniques in insects,” in *Neuromethods*, eds Rogers L. J. Vallortigara G. (New York: Springer), 471–519. 10.1007/978-1-4939-6725-4_15

[B32] Paoli M. Münch D. Haase A. Skoulakis E. Turin L. Galizia C. G. (2017b). Minute impurities contribute significantly to olfactory receptor ligand studies: tales from testing the vibration theory. *Eneuro* 4:2017. 10.1523/ENEURO.0070-17.2017 28670618 PMC5490255

[B33] Paoli M. Galizia G. C. (2021). Olfactory coding in honeybees. *Cell Tissue Res.* 383 35–58. 10.1007/s00441-020-03385-5 33443623 PMC7873095

[B34] Paoli M. Haase A. (2018). “In vivo two-photon imaging of the olfactory system in insects,” in *Olfactory Receptors*, eds Simoes A. G. de Souza F. (New York: Humana Press), 179–219. 10.1007/978-1-4939-8609-5_15 29884947

[B35] Park K. Cork A. (1999). Electrophysiological responses of antennal receptor neurons in female Australian sheep blowflies, Lucilia cuprina, to host odours. *J. Insect Physiol.* 45 85–91. 10.1016/S0022-1910(98)00102-4 12770399

[B36] Piana M. Canfora M. Riani M. (2000). Role of noise in image processing by the human perceptive system. *Phys. Rev. E Stat. Phys. Plasmas, Fluids, Relat. Interdiscip. Topics* 62 1104–1109. 10.1103/PhysRevE.62.1104 11088566

[B37] Sato K. Pellegrino M. Nakagawa T. Nakagawa T. Vosshall L. B. Touhara K. (2008). Insect olfactory receptors are heteromeric ligand-gated ion channels. *Nature* 452 1002–1006. 10.1038/nature06850 18408712

[B38] Schwartzkopff J. (1974). “Mechanoreception,” in *The Physiology of Insecta*, ed Rockstein M. (Amsterdam: Elsevier), 273–352. 10.1016/B978-0-12-591602-8.50013-8

[B39] Suver M. P. Matheson A. M. M. Sarkar S. Damiata M. Schoppik D. Nagel K. I. (2019). Encoding of wind direction by central neurons in *Drosophila*. *Neuron* 102 828–842.e7. 10.1016/j.neuron.2019.03.012 30948249 PMC6533146

[B40] Tuckman H. Kim J. Rangan A. Lei H. Patel M. (2021a). Dynamics of sensory integration of olfactory and mechanical stimuli within the response patterns of moth antennal lobe neurons. *J. Theor. Biol.* 509:110510. 10.1016/j.jtbi.2020.110510 33022286

[B41] Tuckman H. Patel M. Lei H. (2021b). Effects of mechanosensory input on the tracking of pulsatile odor stimuli by moth antennal lobe neurons. *Front. Neurosci.* 15:739730. 10.3389/fnins.2021.739730 34690678 PMC8529024

[B42] Vermeulen A. Rospars J. P. (2004). Why are insect olfactory receptor neurons grouped into sensilla? The teachings of a model investigating the effects of the electrical interaction between neurons on the transepithelial potential and the neuronal transmembrane potential. *Eur. Biophys. J.* 33 633–643. 10.1007/s00249-004-0405-4 15138735

[B43] Vickers N. J. (2000). Mechanisms of animal navigation in odor plumes. *Biol. Bull.* 198 203–212. 10.2307/1542524 10786941

[B44] Wakazono Y. Sakurai T. Terakawa S. (2017). Suppression of ciliary movements by a hypertonic stress in the newt olfactory receptor neuron. *Am. J. Physiol. Cell Physiol.* 313 C371–C379. 10.1152/ajpcell.00243.2016 28684540

[B45] Yorozu S. Wong A. Fischer B. J. Dankert H. Kernan M. J. Kamikouchi A. (2009). Distinct sensory representations of wind and near-field sound in the *Drosophila* brain. *Nature* 458 201–205. 10.1038/nature07843 19279637 PMC2755041

[B46] Zhou C. Franconville R. Vaughan A. G. Robinett C. C. Jayaraman V. Baker B. S. (2015). Central neural circuitry mediating courtship song perception in male *Drosophila*. *Elife* 4:e08477. 10.7554/eLife.08477 26390382 PMC4575990

