# Supplementary material for: Parallel Processing of Olfactory and Mechanosensory Information in the Honey Bee Antennal Lobe
Source: Front Physiol. 2021 Dec 7;12:790453. doi: 10.3389/fphys.2021.790453 (PMC8691435; doi:10.3389/fphys.2021.790453)
Supplement: Supplementary file 1 [file Data_Sheet_1.pdf]

Supplementary Material

1 Tables

Table S1. Statistical results from a repeated-measures ANOVA for each glomerulus between bees and using within-subject factors stimulus type (s) and trial (t). Showing *F* statistics, degrees of freedom (*df*), and probabilities for simple within-subject effects (s, t) and interactions (s×t).

Glo	F_s	df_s	$df_{s,\text{error}}$	p_s	F_t	df_t	$df_{t,\text{error}}$	p_t	$F_{s\times t}$	$df_{s\times t}$	$df_{s\times t,\text{error}}$	$p_{s\times t}$
17	2.80	7	42	0.018	1.46	14	84	0.15	0.89	98	588	0.76
23	7.76	7	42	5.2e-6	0.90	14	84	0.57	0.93	98	588	0.66
25	2.82	7	35	0.020	0.66	14	70	0.81	0.89	98	490	0.76
27	1.27	7	7	0.38	3.26	14	14	0.018	1.11	98	98	0.30
28	29.60	7	42	2.8e-14	4.84	14	84	1.9e-6	2.72	98	588	1.6e-13
29	14.44	7	42	2.2e-9	0.92	14	84	0.55	1.00	98	588	0.48
33	13.15	7	42	8.0e-9	0.79	14	84	0.69	0.93	98	588	0.66
35	1.83	7	35	0.12	1.34	14	70	0.21	1.07	98	490	0.32
36	5.38	7	42	1.9e-4	3.08	14	84	7.0e-4	1.26	98	588	0.060
37	6.24	7	35	8.7e-5	0.98	14	70	0.49	1.04	98	490	0.40
38	3.58	7	42	0.0042	1.06	14	84	0.41	1.16	98	588	0.17
42	3.98	7	42	0.0021	0.85	14	84	0.62	0.97	98	588	0.56
43	0.88	7	7	0.57	1.49	14	14	0.24	0.89	98	98	0.71
47	1.78	7	35	0.13	0.77	14	70	0.71	0.91	98	490	0.71
48	1.86	7	35	0.11	0.55	14	70	0.90	0.77	98	490	0.95
49	2.15	7	42	0.060	0.79	14	84	0.68	1.18	98	588	0.13
60	7.57	7	14	7.2e-4	1.03	14	28	0.46	0.88	98	196	0.76

Table S2. Comparisons between responses to different air flow velocities, that are plotted in **Figure 1G**. Multiple comparison are performed on all trials in all bees. The table is limited to those glomeruli and stimulus pairs that show significant differences according to paired-*t* tests with a Bonferroni correction against type I errors. Columns show the two compared stimulus types, the Glomerulus ID, the mean difference, its standard error, the *t* statistics, the degrees of freedom (*df*), the single test *p* value, and the Bonferroni-corrected *p* value.

Stim1	Stim2	Glo	Mean Diff	Std Error	*t*	*df*	*p*	p_{bonf}
HF	Bkg	23	-2.97	0.67	-4.40	104	2.6e-5	8.7e-4
HF	Bkg	27	4.58	0.34	13.48	29	5.1e-14	2.4e-12
HF	Bkg	28	-3.53	0.56	-6.28	104	7.8e-9	2.9e-7
HF	Bkg	29	7.43	0.51	14.56	104	7.5e-27	3.8e-25
HF	Bkg	33	7.85	0.55	14.38	104	1.8e-26	9.2e-25
HF	Bkg	35	2.49	0.74	3.38	89	0.0011	0.028
HF	Bkg	37	8.12	1.03	7.91	89	6.5e-12	2.9e-10
HF	Bkg	38	3.97	0.59	6.70	104	1.1e-9	4.4e-8
HF	Bkg	42	-8.39	0.98	-8.53	104	1.3e-13	5.8e-12
HF	Bkg	43	-5.57	1.21	-4.59	29	7.9e-5	0.0025
HF	Bkg	49	2.67	0.76	3.51	104	6.7e-4	0.019
HF	Bkg	60	2.87	0.55	5.26	44	4.1e-6	1.4e-4
LF	Bkg	23	-3.52	0.47	-7.53	104	1.9e-11	8.1e-10
LF	Bkg	25	3.10	0.51	6.14	89	2.3e-8	8.3e-7
LF	Bkg	27	1.39	0.37	3.73	29	8.3e-4	0.022
LF	Bkg	28	-3.71	0.55	-6.74	104	9e-10	3.7e-8
LF	Bkg	29	3.71	0.38	9.84	104	1.6e-16	7.4e-15
LF	Bkg	33	3.11	0.41	7.53	104	1.9e-11	8.1e-10
LF	Bkg	36	-4.10	0.64	-6.46	104	3.4e-09	1.3e-7
LF	Bkg	37	3.45	0.88	3.91	89	1.8e-4	0.0053
LF	Bkg	42	-7.81	0.78	-10.02	104	6.2e-17	3e-15
LF	Bkg	43	-6.58	1.47	-4.49	29	1.0e-4	0.0032
HF	LF	27	3.19	0.50	6.44	29	4.9e-7	1.7e-5
HF	LF	29	3.73	0.59	6.33	104	6.4e-9	2.4e-7
HF	LF	33	4.74	0.42	11.34	104	6.9e-20	3.4e-18
HF	LF	38	2.75	0.83	3.31	104	0.0013	0.032
HF	LF	49	3.40	0.86	3.94	104	1.5e-4	0.0045

Table S3. Comparisons between responses to air stimuli with and without odor, that are plotted in **Figure 2F**. Multiple comparison are performed on all trials in all bees. The table is limited to those glomeruli and stimulus pairs that show significant differences according to paired-*t* tests with a Bonferroni correction against type I errors. Columns show the two compared stimulus types, the Glomerulus ID, the mean difference, its standard error, the *t* statistics, the degrees of freedom (*df*), the single test *p* value, and the Bonferroni-corrected *p* value.

Stim1	Stim2	Glo	Mean Diff	Std Error	*t*	*df*	*p*	p_{bonf}
HF+3Hex	HF	28	15.73	0.79	-20.02	104	1.9e-37	6.3e-36
HF+3Hex	HF	29	-2.04	0.58	3.52	104	6.4e-4	0.018
HF+3Hex	HF	36	11.17	1.09	-10.23	104	2.1e-17	6.6e-16
LF+3Hex	LF	17	-2.33	0.53	4.38	104	2.9e-5	8.3e-4
LF+3Hex	LF	23	-3.79	0.57	6.69	104	1.1e-9	3.4e-8
LF+3Hex	LF	28	12.69	0.76	-16.59	104	5.6e-31	1.9e-29
LF+3Hex	LF	36	8.84	0.94	-9.40	104	1.5e-15	4.6e-14
LF+3Hex	LF	48	-1.69	0.48	3.52	89	6.8e-4	0.018

Table S4. Comparisons between responses to a waggling winglet, that are plotted in **Figure 3H**. Multiple comparison are performed on all trials in all bees. The table is limited to those glomeruli that show significant differences between stimulus response and background activity according to paired-*t* tests with a Bonferroni correction against type I errors. Columns show the two compared stimulus types, the Glomerulus ID, the mean difference, its standard error, the *t* statistics, the degrees of freedom (*df*), the single test *p* value, and the Bonferroni-corrected *p* value.

Stim1	Stim2	Glo	Mean Diff	Std Error	*t*	*df*	*p*	p_{bonf}
Wag	Bkg	17	2.50	0.57	4.43	104	2.4e-5	0.0001y9
Wag	Bkg	23	3.82	0.37	10.31	104	1.4e-17	2.3e-16
Wag	Bkg	25	-1.41	0.33	-4.25	89	5.3e-5	3.7e-4
Wag	Bkg	27	2.66	0.59	4.53	29	9.2e-5	5.5e-4
Wag	Bkg	29	1.77	0.39	4.50	104	1.8e-5	1.8e-4
Wag	Bkg	33	1.62	0.33	4.83	104	4.7e-6	5.2e-05
Wag	Bkg	35	1.98	0.52	3.82	89	2.4e-4	0.0012
Wag	Bkg	36	-1.55	0.47	-3.27	104	0.0015	0.0058
Wag	Bkg	37	4.35	0.84	5.20	89	1.2e-6	1.5e-5
Wag	Bkg	38	2.66	0.50	5.26	104	7.6e-7	9.9e-6
Wag	Bkg	42	-4.59	0.65	-7.08	104	1.7e-10	2.4e-9
Wag	Bkg	43	3.23	0.94	3.42	29	0.0019	0.0058
Wag	Bkg	47	1.66	0.57	2.90	89	0.0047	0.0094
Wag	Bkg	48	4.00	0.48	8.34	89	8.5e-13	1.3e-11
Wag	Bkg	49	2.31	0.51	4.48	104	1.9e-5	1.8e-4
Wag	Bkg	60	4.68	0.45	10.4	44	2e-13	3.2e-12

Table S5. Comparisons between responses to air flow with and without waggling modulation, that are plotted in **Figure 3I**. Multiple comparison are performed on all trials in all bees. The table is limited to those glomeruli that show significant different responses to air flow stimuli with and without waggling according to paired-*t* tests with a Bonferroni correction against type I errors. Columns show the two compared stimulus types, the Glomerulus ID, the mean difference, its standard error, the *t* statistics, the degrees of freedom, the single test *p* value, and the Bonferroni-corrected *p* value.

Stim1	Stim2	Glo	Mean Diff	Std Error	*t*	*df*	*p*	p_{bonf}
HF+Wag	HF	17	-1.75	0.51	3.45	104	8.0e-4	0.018
HF+Wag	HF	23	3.42	0.62	-5.48	104	3e-7	9.4e-6
HF+Wag	HF	28	2.50	0.69	-3.6	104	4.9e-4	0.012
HF+Wag	HF	36	2.77	0.62	-4.44	104	2.2e-5	6.5e-4
LF+Wag	LF	23	6.28	0.64	-9.85	104	1.4e-16	4.8e-15
LF+Wag	LF	25	-4.08	0.63	6.48	89	4.9e-9	1.6e-7
LF+Wag	LF	28	2.66	0.67	-4.00	104	1.2e-4	0.0031
LF+Wag	LF	43	8.90	2.03	-4.39	29	1.4e-4	0.0035
LF+Wag	LF	47	3.26	0.80	-4.07	89	1.0e-4	0.0028
LF+Wag	LF	48	2.38	0.51	-4.72	89	8.8e-6	2.6e-4
LF+Wag	LF	49	3.09	0.77	-4.03	104	1.1e-4	0.0028
LF+Wag	LF	60	4.42	0.52	-8.42	44	1e-10	3.3e-9

2 Figures

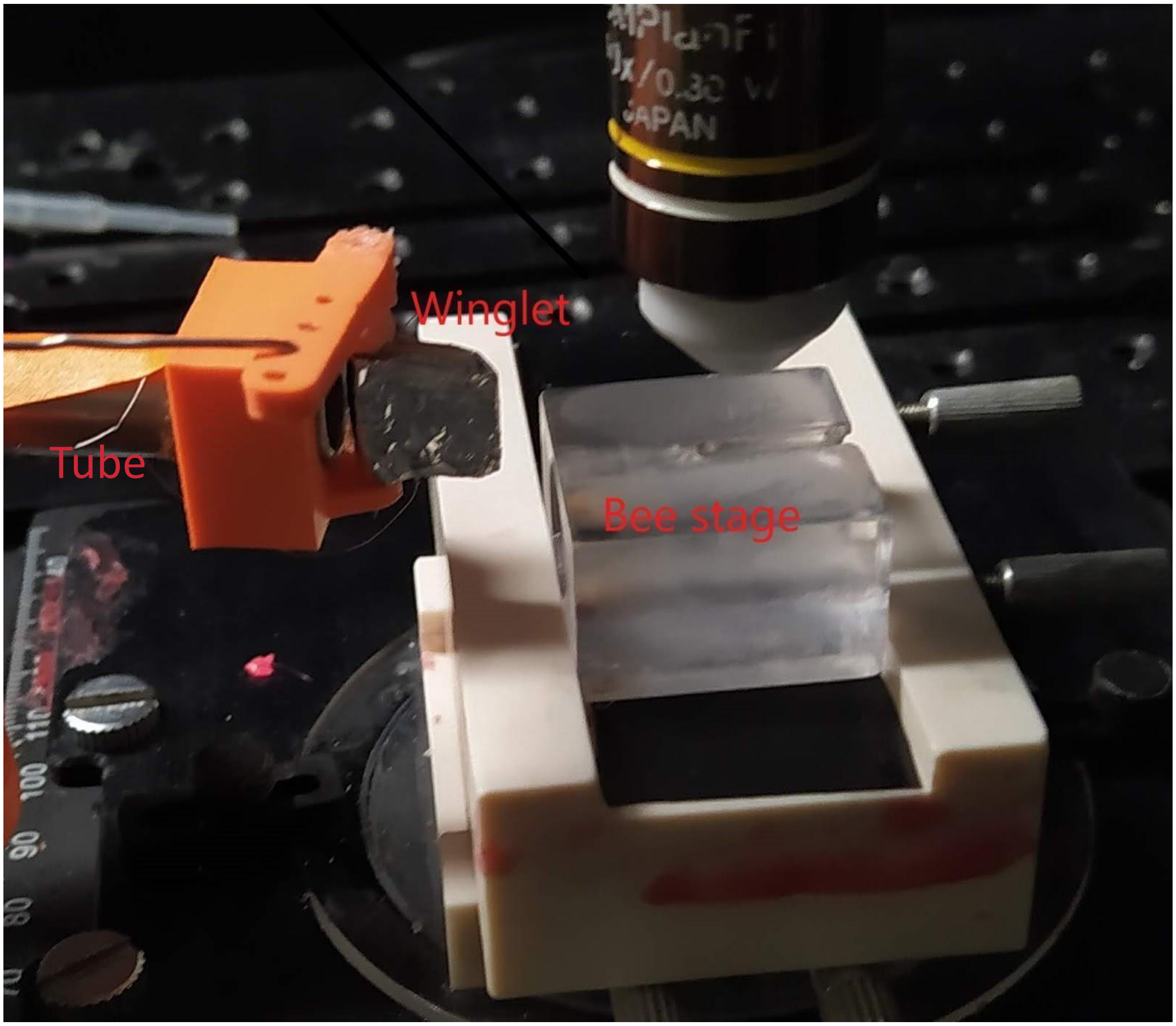

Supplementary Figure 1. [Stimulus generator]. Bees are mounted on the stage facing the winglet. The airflow is directed through the steel tube straight toward the antennae. For the waggle dance simulation, the winglet is activated by a motor whose speed is detected through a rotary encoder.

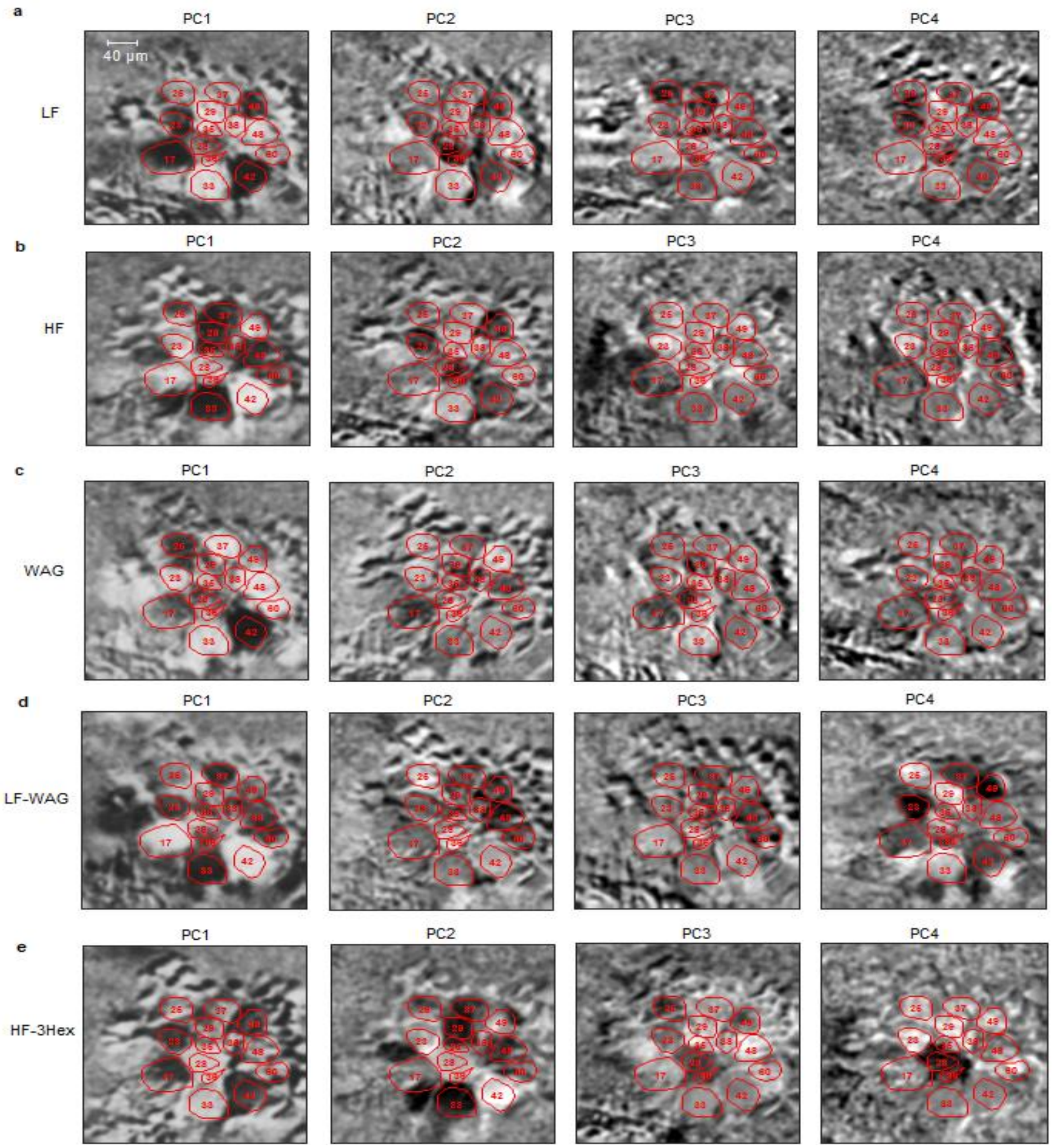

Supplementary Figure 2. [Principal components highlight glomeruli with the greatest signal variance during a stimulus]. The frames of a stimulus period were averaged over trials, normalized, and converted into vectors. A PCA was then performed with pixels as variables and time frames as observations. The first PC is the variance-maximizing projection of stimulus-related signals, spontaneous activity, and sample movements (Strauch et al., 2013). The strongest glomerular responses show high eigenvalues in the first principal component. Signals in the periphery are due to highly active neuronal somata and sample motion. The maps evidence the broad involvements of several glomeruli to stimuli encoding. (**a**) Glomerular pattern elicited by LF stimulation. (**b**) Glomerular pattern elicited by HF stimulation. (**c**) Glomerular pattern elicited by waggling stimulation. (**d**) Glomerular pattern elicited by LF airstream modulated by waggling. € Glomerular pattern elicited by the odor 3-Hexanol.

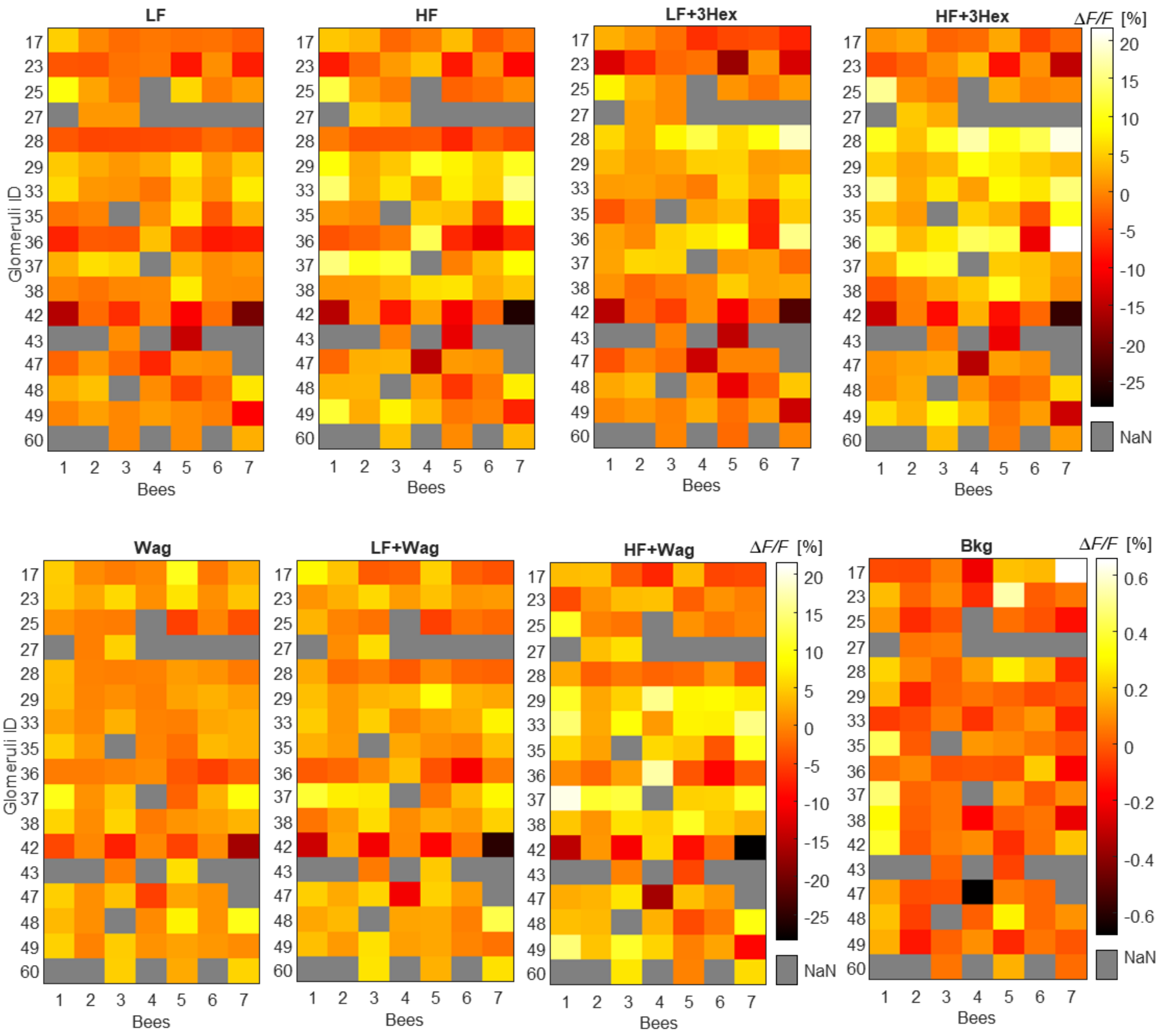

Supplementary Figure 3. [**Maps of the glomerular responses to the different stimuli, for each recorded bee].** Shown is the trial- and time-averaged activity from 2 - 4 s after stimulus onset. Grey areas mark glomeruli that could not be recorded. The color scale is identical for the 7 stimuli: Low flux (LF), High flux (LF), Low flux + 3-Hexanol (LF+3Hex), How flux + 3-Hexanol (HF+3Hex), Waggling (Wag), Low flux + Waggling (LF+Wag), How flux + Waggling (HF+Wag), for the background activity (Bkg) the color scale is different, to highlight the small oscillations in the background signal.

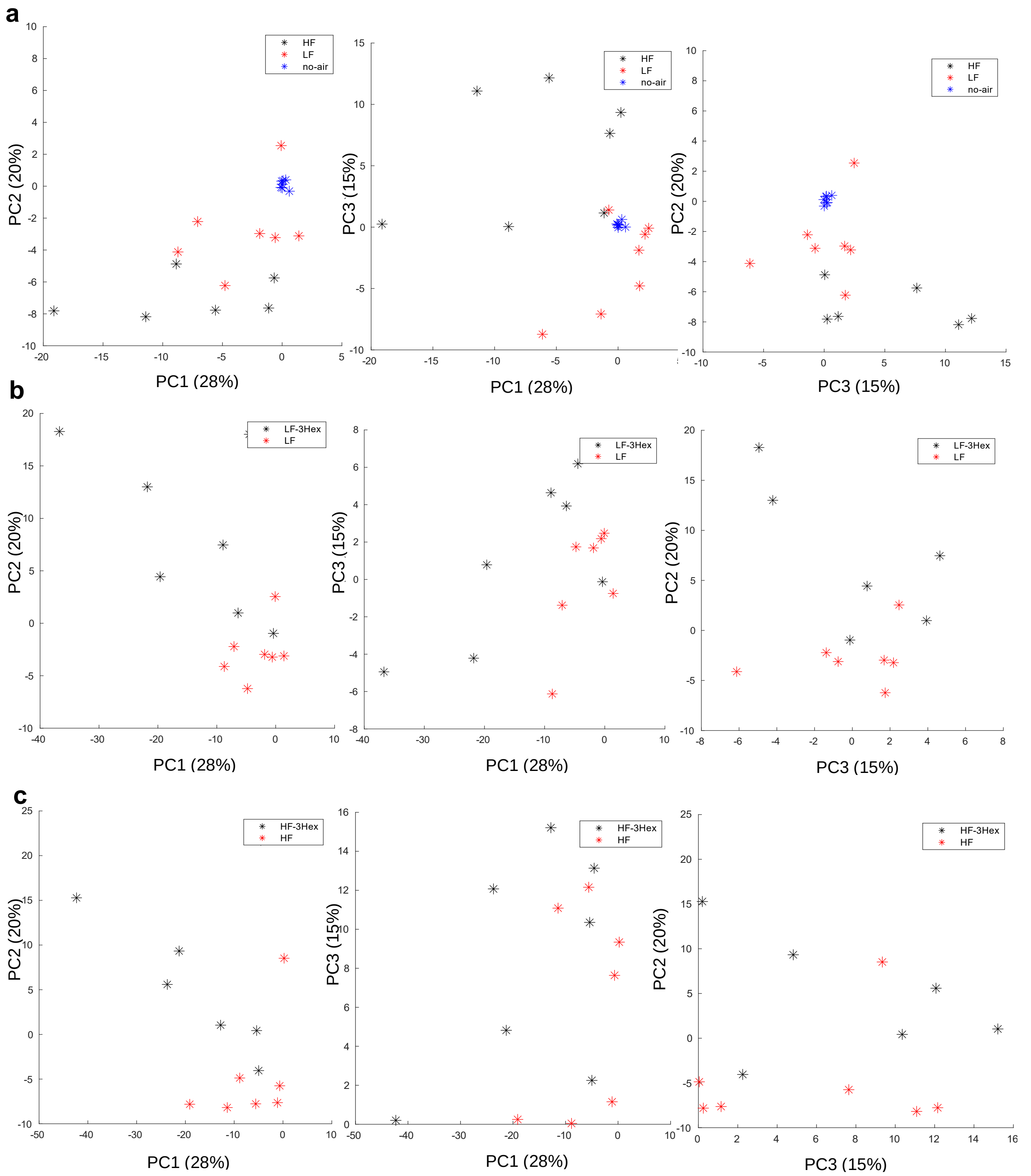

Supplementary Figure 4. [PCA of the glomerular response space. Each individual bee response to the stimuli is described in terms of the 3 first PCs]. **(a)** Comparisons between LF-HF+no-air stimuli. A statistical energy test (Aslan and Zech, 2005) gives HF *vs.* LF ($\varphi(7) = 22.0$, $p = 0.022$), no-air *vs.* LF ($\varphi(7) = 18.9$, $p = 0.001$), no-air *vs.* HF ($\varphi(7) = 50.1$, $p = 0.001$). **(b)** Comparisons between LF and LF-3Hex ($\varphi(7) = 51.3$, $p = 0.004$). **(c)** Comparisons between HF and HF-3Hex ($\varphi(7) = 42.3$, $p = 0.037$). All differences are significant including the Bonferroni correction for type I errors.

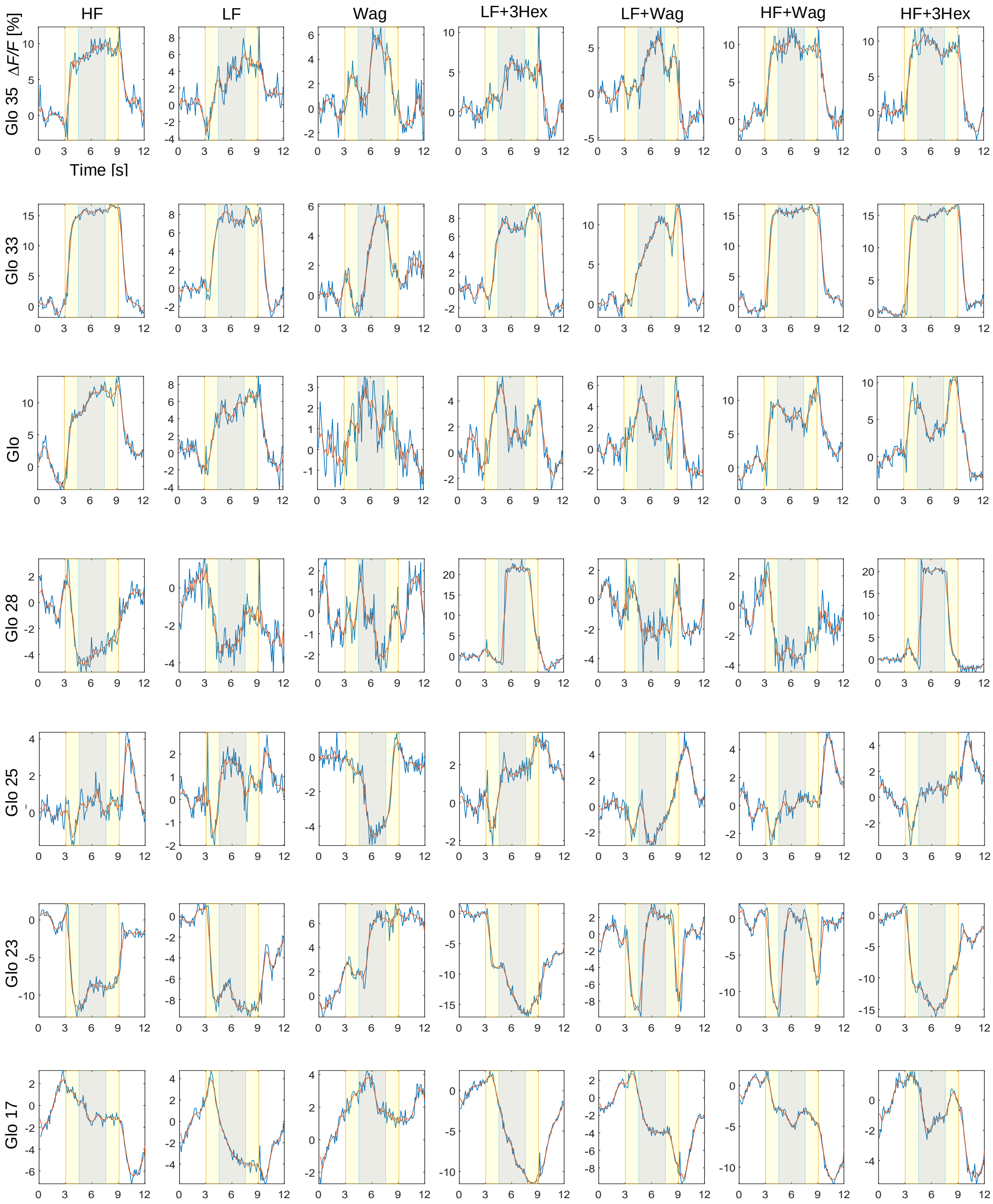

Supplementary Figure 5. [Complete set of stimuli of a representative bee (Glo 17 - Glo 35)]. Rows show individual glomeruli, columns the different stimuli. Blue lines represent the response averaged over all 15 trials; the red line shows the low-pass filtered response. Yellow areas highlight the airstream stimulus period, the grey area the additional odor or waggle stimuli. The stimulus order is the same as provided during the experiment. Signal intensity is expressed as $\Delta F/F$ [%].

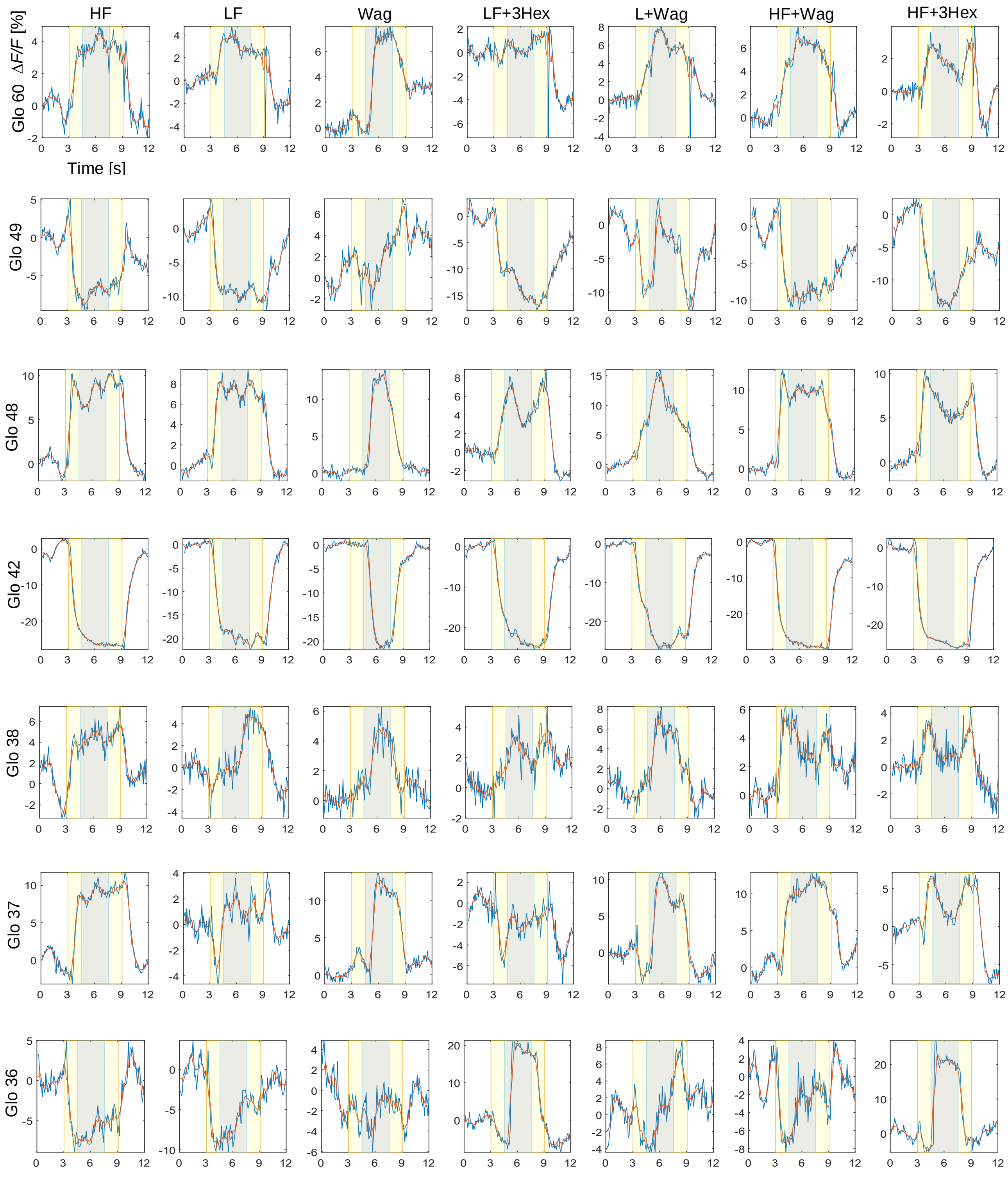

Supplementary Figure 6. [Complete set of stimuli of a representative bee (Glo 36-Glo 60)]. Rows show individual glomeruli, columns the different stimuli. Blue lines represent the response averaged over all 15 trials, the red line shows the low-pass filtered response. Yellow areas highlight the airstream stimulus period, the grey area the additional odor or waggle stimuli. The stimulus order is the same as provided during the experiment. The signal intensity is expressed as Δ*F/F* [%].

Supplementary material references

Aslan, B., Zech, G. (2005). Statistical energy as a tool for binning-free, multivariate goodness-of-fit tests, two-sample comparison and unfolding. Nucl Instrum Methods Phys Res. 537: 626–36. https://doi.org/10.1016/j.nima.2004.08.071

Strauch, M., Rein, J., Lutz, C., Galizia, C.G. (2013). Signal extraction from movies of honeybee brain activity: the ImageBee plugin for KNIME. BMC Bioinformatics.14(Suppl 18): S4. https://doi.org/10.1186/1471-2105-14-S18-S4
